# Correlation between Androgen Receptor Expression and Immunohistochemistry Type as Prognostic Factors in a Cohort of Breast Cancer Patients: Result from a Single-Center, Cross Sectional Study

**DOI:** 10.3390/healthcare9030277

**Published:** 2021-03-03

**Authors:** Irina Niță, Cornelia Nițipir, Ștefania Andreea Toma, Alexandra Maria Limbău, Edvina Pîrvu, Ioana Anca Bădărău, Ioana Suciu, George Suciu, Loredana Sabina Cornelia Manolescu

**Affiliations:** 1 Department of Microbiology, Parasitology and Virology, Faculty of Medicine, Midwifery and Nursing, Carol Davila University of Medicine and Pharmacy, 050474 Bucharest, Romania; nitipir2003@yahoo.com (C.N.); ancab52@yahoo.com (I.A.B.); 2 Medical Oncology Department, Elias University Emergency Hospital, 011461 Bucharest, Romania; 3 Medical Oncology Department, Ponderas Academic Hospital, 014142 Bucharest, Romania; stefaniaandreeachivu@yahoo.com; 4 Dermatology Department, Municipal Hospital Curtea de Argeș, 115300 Curtea de Argeș, Romania; alexandra_021286@yahoo.fr; 5 Medical Oncology Department, Clinical Hospital “Colţea”, 030167 Bucharest, Romania; edvinapirvu@gmail.com; 6 R&D Department, BEIA Consult International, 010158 Bucharest, Romania; ioana.suciu@beia.ro (I.S.); george@beia.ro (G.S.)

**Keywords:** androgen receptor, breast cancer, immunohistochemistry, overall survival, progression free survival

## Abstract

Background: We investigated the correlation between the androgen receptor (AR) and immunohistochemistry (IHC) as a prognostic factor in breast cancer (BC). AR is expressed in 60–80% of BC. Methods: We evaluated the prognostic values of AR expression among 143 patients with BC for 36 months. The protocol was amended to measure androgen, estrogen and progesterone receptor expression by IHC and the percentage of hormone positive nuclei was quantified. We determined and quantified the Her2/neu status using IHC and in situ hybridization. The methodology consisted in using a Kaplan–Meier analysis and restricted mean survival time up to 36 months. The principal endpoints of the study were overall survival (OS) and progression free survival (PFS). Results: 57% of patients (*n* = 82) from our group had AR+ (≥ 1%). Patients with AR+ had better OS, 35.50 vs. 33.40 months, with *p* = 0.027. Moreover, PFS was prolonged for patients AR+, 32.60 vs. 30.50 months, with *p* = 0.38. Triple negative breast cancer (TNBC) patients had lower OS and no difference was observed for PFS. Conclusions: Both OS and PFS were favorably influenced by the presence of AR. TNBC had worse outcomes compared with patients with hormonal or/and Her 2/neu positive disease in terms of OS.

## 1. Introduction

Breast cancer (BC) is the most frequent type of cancer among women worldwide. In this disease, although many/ most of the main risk factors are known, as well as the pathogenic pathways, the mechanism of metastasis and therapeutic strategies, death related cancer remains very high. The widespread possibilities of neo-adjuvant or adjuvant therapy have reduced mortality, but there are some patients over treated and others undertreated. For a complete characterization of a breast cancer, American Joint Committee on Cancer (AJCC) and World Health Organization (WHO) guidelines require classification of malignant tumors (TNM) and a minimal histo-pathological report that must include the grade of differentiation according to Nottingham scale and an immune-histo-chemical panel for prognostic and predictive factors for molecular classification in luminal or non-luminal subtypes. Those are hormone receptor expression, estrogen (ER) and progesterone (PR), proliferation index measured using antigen Ki 67 and the HER2neu oncogene status. Even with this stratification that allows an increasing specificity for certain treatments, novel expensive models of appreciating the opportunity of treatment in selected population (Oncotype Dx, MammaPrint, etc.), we consider that it would be of great value to have supplementary prognostic factors, economically reliable that may help the clinicians to personalize therapies [1,2,3]. 

By definition, a prognostic factor provides information on clinical outcome at the moment of diagnosis. Such markers are predominantly referred to the evolution of the tumor and can provide information related to growth, invasion and metastatic potential. Mammographic features, pathologic aspects or receptor status are prognostic factors used in clinical routine [4,5,6].

### 1.1. Androgen Receptor

Estrogen, progesterone and androgen receptor (AR) are sex steroid hormone receptors, which have a primordial role in development and spread of BC. Even more, estrogen blockade using selective estrogen modulators and aromatase inhibitors are fundamental therapeutic strategies for BC patients [7,8].

The case of a patient where a receptor for androgen is identified and expressed in BC is very interesting considering the fact that the tumor is predominantly estrogen dependent. It has been noted that 70–90% of BC patients overexpress the AR with several studies indicating that AR might be a predictive or prognostic factor and a drug target in BC [9].

Taking into account the heterogeneity of the disease, that could indicate the lack of response to hormonal treatments in some BC expressing hormones, the AR could turn up to be as a new marker and potential new therapeutic target, following the possibilities opened up by the AR inhibitors approved for the treatment of prostate cancer [10].

An important aspect is to understand the locations of the AR and its functional significance to better reveal its complex role in BC. There is increasing evidence to suggest the existence of membrane ARs, however their exact mechanism and structure have still to be elucidated. Debate surrounds whether sex steroid receptors can be located in the cell membrane or localized at the cytoplasmic membrane. Some studies suggest that membrane AR is the classical AR receptor, but a modification such as palmitoylation enables translocation to the membrane [11,12].

Despite the location and the cell mechanism of AR, an important aspect is to demonstrate the impact of blood androgens. Studies reported in breast cancers a relation between mediation of gene transcription by conjugated testosterone (that does not cross the cell membrane) and non-conjugated ligand. It was discovered that in an AR negative cell line a large proportion of the genes modified by testosterone were also affected by the conjugated form of testosterone. However, in AR positive cells a significant number of genes were induced with testosterone and they were not observed with conjugated ligand treatment [13,14].

The fact that AR has a part to play in the development of BC seems to be indisputable and brought up by preclinical and clinical findings, even though the influence of circulating androgens in the progression of BC is not fully understood. Although the BC diagnosis is very accurate using the IHC protocols, there are some cases, especially the triple negatives which are challenging. Sry-related HMg-Box gene 10, GATA-binding protein 3, gross cystic disease fluid protein 15, mammaglobin and AR are useful IHC markers which may help on differential diagnosis of a metastatic disease [15,16].

AR is one of the members of the steroid receptor family, which includes estrogen and progesterone receptors. It is expressed in both normal and malignant tissue, in about 60–80% of breast carcinomas, in different types of histology. While the estrogen receptor (ER), progesterone receptor (PR) and human epidermal growth factor (Her 2/neu) are routinely assessed in BC for helping in prognosis and treatment decision- making, this is not the case for AR. Recently, clinical research has shifted the interest to AR as a possible therapeutic option in BC treatment, due to the high degree of presence on the tumor cells and lack of therapeutic drugs [17,18,19].

Preclinical and clinical data showed that the effect of AR is different for each hormonal status of cancer cells. In ER positive cells, in vitro models showed that AR has an antagonistic effect to the estrogens. This may occur through competition between ER and AR for binding sites, at estrogen response elements, or through competition for transcriptional co- regulators. In terms of survival, population- based studies support the hypothesis that there is an inverse association between tumor AR expression and mortality in women with ER positive BC [9,14].

Preclinical studies have surmised that AR may enact its protective role by blocking ERα gene transcription, however, other studies in triple-negative and apocrine BC indicate that AR may act as a pseudo- ERα in this setting. There has also been some confounding clinical data in which AR positivity indicates lack of complete clinical response to neo-adjuvant chemotherapy in contrast to patients who are negative for both AR and Erα [20,21,22,23].

The oncoprotein Her 2/ neu and the AR are positive in approximate 60% of BC. In this population, about 10% of the tumors with co-expression of those receptors were associated with less aggressive tumors, (higher expression of ER and progesterone, earlier clinical stage, lower ki67 and smaller tumor size), so with better outcome in terms of survival [14,24].

AR is expressed in about 10–35% of triple negative breast cancer (TNBC), which does not express ER, PR and Her2/neu and tends to be more aggressive than other subtypes of BC. In this group of patients, the positive of AR is associated with better PFS and OS. This response was also seen in patients in whom the pathological complete response was not present after neo-adjuvant chemotherapy [25].

In addition to the diagnostic and therapeutic side, targeting AR represented an important aspect to take in consideration. For instance, the interest was carried on molecules which may block the receptors or the interaction with AR pathways that cause cell proliferation. Bandini et al. suggested that a specific uptake can help the purpose to customize miRNAs as therapeutics alone or in combination with other oncologic treatments [26].

In recent years there has been a growing interest in targeting the AR in BC either by using AR agonists such as enobosarm or by antagonizing AR actions using drugs primarily developed as therapeutic agents for prostate cancer, specifically bicalutamide, enzalutamide and the CYP17 inhibitor, abiraterone. Results from these trials have been varied with better outcomes reported in AR positive triple negative tumors [27,28,29,30].

In the following subsections, we aim to categorize BC from the standpoint of the molecular stratification based on the receptors displayed on the tumor cell surface. This study was designed to evaluate the use of AR and IHC subtypes as prognostic markers in early and advanced BC cohorts. Our aim is to demonstrate the importance of routinely assessing the AR, helping the clinicians to have a good overview regarding the prognosis and to deliver the best treatment choice for each case.

### 1.2. Immunohistochemistry

IHC is valuable to identify the tumor lineage in patients with poorly differentiated cancer, to suggest the specific tumor type and the origin of the tissue. The antibodies used in this technique are directed to specific cell components or cell products including enzymes, hormones, normal tissues components or tumor markers. Overall, IHC offers clinicians’ aspects related to the tumor, but also information about the therapeutic possibilities [31,32].

In BC patients, the IHC testing provides information on the status of hormone receptors. ER and PR evaluation are essential for all newly diagnosed cases of breast cancer, and when applicable, for recurrent/metastatic ones. Positive ER and PR expression is associated with longer disease-free status and overall survival and is an indicator of responsiveness to endocrine therapy [1,2].

For Her2/neu (ISH) for IHC 1+ or 2+ is used in conjunction with in situ hybridization to ensure a description as accurate as possible. The results of this analysis are useful for assessing the prognosis of patients, but also for delivering targeted molecular treatment [33].

The IHC classification correlates well with intrinsic gene expression microarray categorization: ER/PR+, Her2− with Luminal A; ER/PR+, Her2+ or / and Ki 67 > 20% with Luminal B; ER/PR− Her2+ and ER/PR−.Her2− with triple negative/basal-like tumors. Apart from lending itself to subtype analyses of tumor when fresh tissue is not available, the IHC classification has prognostic and therapeutic implications, is inexpensive and readily available [7]. 

## 2. Materials and Methods

We conducted a retrospective, observational study on the early and locally advanced BC patients treated in Elias Emergency Hospital Bucharest, Romania, between January 2014 and December 2019. Our research was carried out with the approval and in accordance with the guidelines of the local Ethics Committee. All the procedures in the study respect the ethical standards in the Helsinki Declaration, and the protocol was approved by the Ethics Committee with the code 7423/2018.

One hundred and forty-three patients newly diagnosed with BC without metastasis disease were recruited in our hospital and submitted in our study. The mean age was 52 (range 27–78, SD = 12).

Eligible patients were ages 18 years or older with an Eastern Cooperative Oncology Group status of 0 or 1, unilateral breast tumor, absence of pregnancy in the last 6 months and locally advanced BC. From this group of patients, we excluded the Human Immunodeficiency Virus (HIV) positive, Venereal Disease Research Laboratory (VDRL) positive or infection with B or C hepatic viruses. We also did not enroll patients who had abnormalities in the Pap smear test, until the histo-pathological confirmation of the benign result or had been diagnosed with cervical cancer [34,35,36].

All patients had IHC assay with staining for ER, PR, AR and for Her 2/neu. A cut-off for positivity of 1% positively stained nuclei was applied for ER and PR. Regarding the Her 2/neu status initial with IHC and, for the samples with 1 or 2+, we performed fluorescence ISH to confirm the status.

Tumor staging was performed clinically or pathologically (if surgery was performed before chemotherapy) using TNM staging. In our study only 4% of patients were eligible to receive only hormonal therapy after surgery; most of the patients were candidates for chemotherapy according to their stage or pathological features.

The chemotherapy schedules were delivered under international guidance consultation. 47% (*n* = 68) of patients were not eligible for surgery and had to receive neo-adjuvant chemotherapy. Subsequently, for approximately 80% of those patients the surgeon performed lumpectomy and after that the patients received radiotherapy.

The dose intensity was an important aspect for respecting the schedule. Patients were always instructed before treatment initiation about the importance of following the schedule and all the efforts were made to avoid delay. However, in 7 patients it was necessary to discontinue the chemotherapy with taxanes due to anaphylactic reactions and in other 7 patients delay of chemotherapy cycle and dose reduction due to febrile neutropenia was necessary. Primary prophylaxis of neutropenia fever was always preferred in the presence of intermediate overall risk after taking into consideration all the risk factors.

### 2.1. AR Assessment

**Figure 1:**
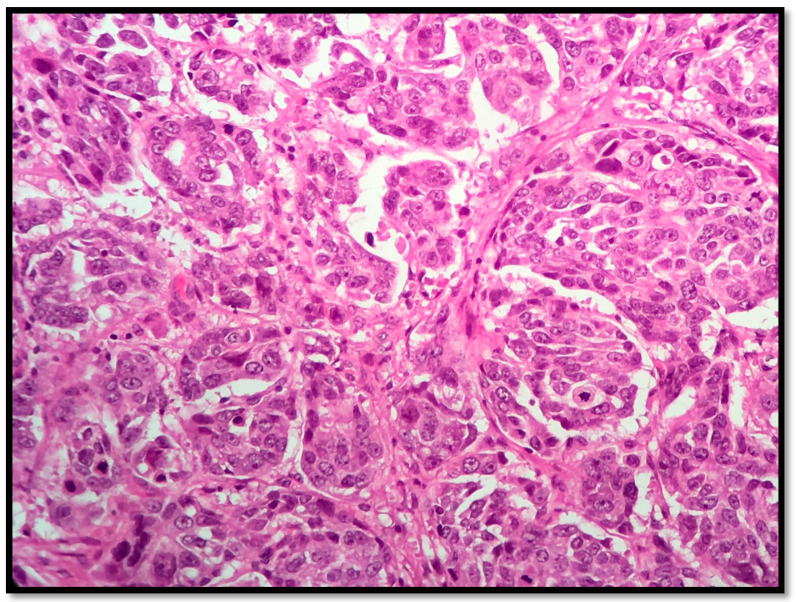
HE, 20 × NST (invasive ductal carcinoma), G2 (mitoses-2, tubule formation-2, nuclear pleomorphism-2).

The samples were obtained from either needle core biopsy or radical mastectomy or lumpectomy, both before chemotherapy. Then they were fixed in 10% buffered formalin, paraffin embedded and stained with Hematoxylin-Eosin for histo-pathological examination, Figure 1. All available hematoxylin and eosin (HE)-stained slides were reviewed. For immunohistochemical studies, the Tissue microarray (TMA) blocks containing representative tumor areas were selected for immunohistochemical stains from all patients, were cut at 3μm thick sections, placed on slides, deparaffinized in xylene and hydrated in a decreasing ethanol series. Immunohistochemical sections were evaluated by both visual and image analysis techniques.

Nuclear staining was evaluated on whole sections by IHC by using monoclonal mouse anti human AR antibody (Dako, AR441 antibody). Following assessment of tumor invasiveness using hematoxylin and eosin-stained slides, a semi-quantitative scoring of AR fractions (0, 1–10%, 11–50%, 51–75%, and 76–100%) of positively stained nuclei, irrespective of nuclear staining intensity, was performed. Nuclear staining in tumor cells was considered positive. Although the activated AR has membrane localization, in our study we assessed 110 kDa protein using IHC methods in order to increase the sensitivity of the AR detection method.

**Figure 2:**
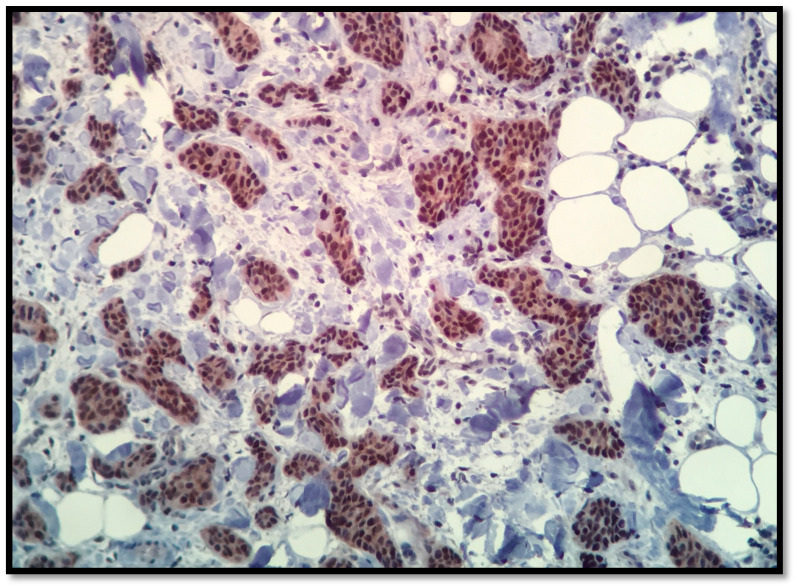
AR, 20×, 90% positivity in NST (invasive ductal) breast carcinoma.

In the evaluation and quantification of positively immune-stained cells, the most prevalent method that we used was the manual counting performed by the pathologist using a conventional microscope. We measured the cellular density of the images with > 100 positive cells/image, Figure 2.

For category variables, the absolute frequencies (number for each category out of the total number) and the relative ones (percentages) were calculated. We used bar plots and dot charts for graphical representation. For continuous (interval) variables, the following were measured: central tendency (arithmetic mean, median), tendency to variability (standard deviation / variance, IQR—inter quartile range the difference between the quartile of 75 and that of 25), deviation of the symmetry distribution (skewness-normal values 0 for Gaussian distribution) and peak of distribution (kurtosis). We also tested the deviation from the normal distribution by means of the Shapiro-Wilk test of normality.

Immune-histo-chemical evaluation was performed according to the percentage and intensity of tumor cells with brown nuclear staining. The intensity of staining was graded semi quantitatively as mild, moderate and strong. Tumors with nuclear staining of 1% or more were accepted as AR-positive, while tumors with AR were scored by a dedicated pathologist, blinded for clinical data, and were considered positive in case of staining in at least 1% of fractions of tumor cells, consistently with most recent studies and specified in a statistical plan [37,38].

### 2.2. Her2 Neu, ER and PR Assessment 

**Figure 3:**
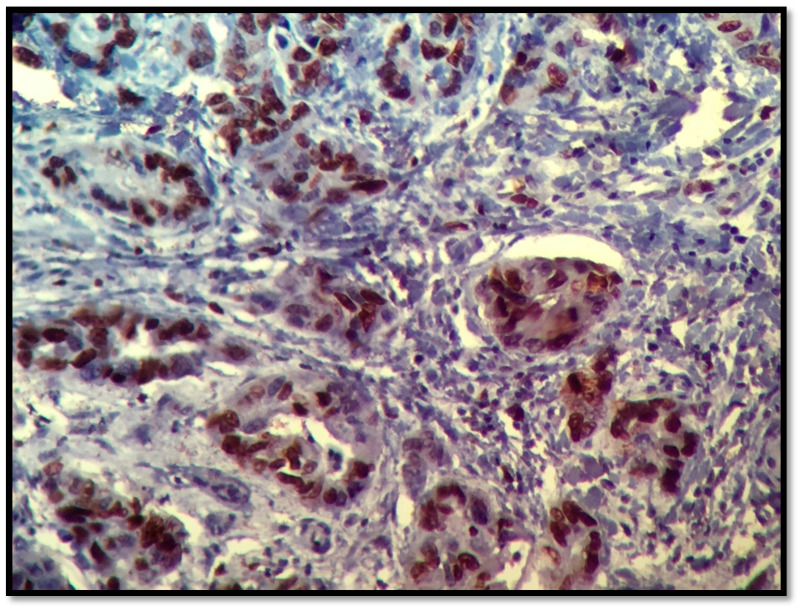
ER, 40×, 80%.

**Figure 4:**
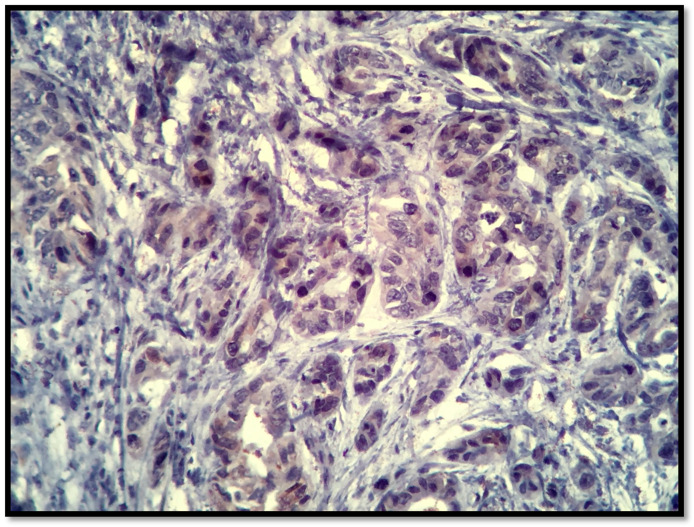
PR, 40×, 1%.

**Figure 5:**
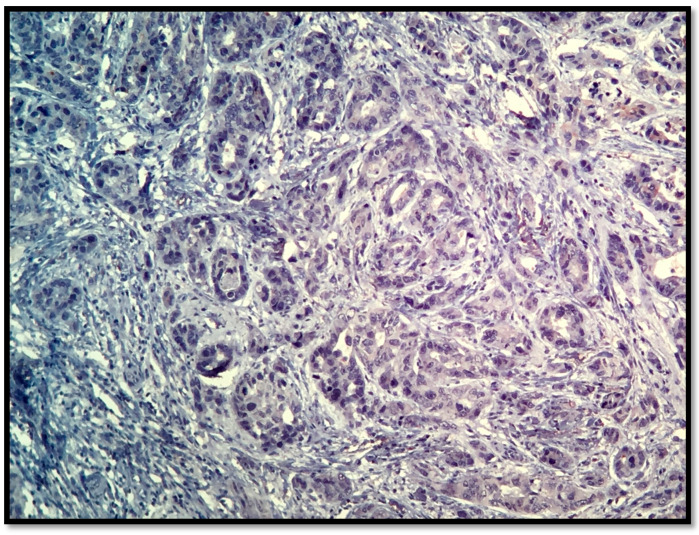
HER2neu, 40×, negative.

**Figure 6:**
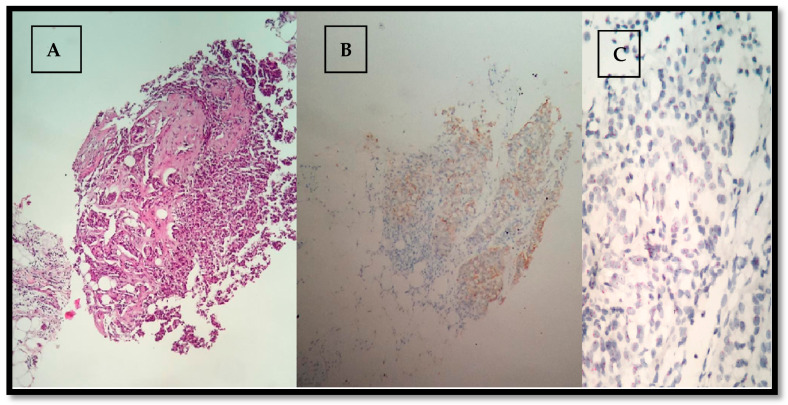
(**A**) HE, 20×, invasive ductal carcinoma (NST); (**B**) HER2neu IHC ++, 20×; (**C**) FISH Her2/neu: chromosome 17 > 2, 40×.

ER, Figure 3, and PR results, Figure 4, were obtained from the histological result having been processed in Accredited Laboratories of Pathology from our city. The ER assay clone used was 1D5, the PR assay clone was PgR636 and the detection system was a polymer. IHC staining permits the detection and localization of ER/PR within sections from formalin-fixed, paraffin-embedded tissues. Staining of > 5% of tumor cell nuclei is considered positive. Staining of 1% to 5% of tumor cell nuclei was considered borderline. Staining of < 1% of tumor cell nuclei was considered negative. Among patients with Her2/neu 2+, the fluorescence in situ hybridization (FISH) or dual in situ hybridization (DISH) method was performed to determine whether the patient was Her2/neu positive or negative, Figure 5 and Figure 6.

Data regarding clinical examination with local evaluation, imagistic assessment, histopathologic and IHC exam were collected at baseline and reviewed at each subsequent visit. Follow-up visits were scheduled every three months and all patients were followed for a maximum of 36 months.

### 2.3. Study Methods

Submission of tumor tissues for AR screening was allowed for the first sample, either core biopsy or surgical intervention. All the samples were collected before any cancer treatment. The IHC assay was performed for ER, PR and her2/neu in different laboratories, under the international protocols. After the cancer confirmation the patients received chemotherapy or hormonal according to clinical stage. The evolution and response were assessed with clinical examination and CT scan every 12 weeks for the first 12 months, then every 24 weeks for the second year and then annually. The disease progression defined by the guidelines Response Evaluation Criteria in Solid Tumors (RECIST) version 1.1 was requested to objectify progressive disease.

### 2.4. Statistical Analysis and Study End Points

For the statistical analysis we had two principal end points: OS and PFS considering androgen receptors and IHC aspects. OS was measured from the date of surgery or biopsy to the date of death or up to 36 months. PFS was measured from the date of surgery or biopsy until the first local or distant recurrence.

For statistical analysis we used the R program, version 4.0.2 (R Foundation for Statistical Computing, Vienna, Austria) and survminer package. The sensitivity level was 95%, with *p* < 0.05, considered statistically significant.

Our research was carried out with the approval and in accordance with the guidelines of the local Ethics Committee. All the procedures in the study respect the ethical standards in the Helsinki Declaration, and the protocol was approved by the Ethics Committee with the code 7423/2018 and 223/2019.

Both OS and PFS were measured using restricted mean survival time (RMST). RMST quantifies the time until the event occurs, illustrated survival functions, and conventionally reported an alternative to hazard ratios to express the magnitude of the treatment effect when comparing between groups. Due to this short follow-up period, 36 months, the median survival could not be calculated [39].

## 3. Results

For the IHC aspects, we shared our groups of patients into four categories regarding the status of ER, PR and her 2/neu.:

- Group I: triple negative breast cancer (TNBC) (*n* = 39)
- Group II: hormonal receptors negative, Her 2/neu positive (*n* = 22)
- Group III: hormonal receptors positive, Her 2/neu negative (*n* = 68)
- Group IV: hormonal receptors positive, Her 2/neu positive (*n* = 14).

We divided the statistical analysis into two chapters according to the established endpoints, OS and PFS, considering the androgen receptors and the IHC results.

Considering the ≥ 1% cut-off, AR was present in 57% of patients (*n* = 89) on the entire group of patients, Table 1.

### 3.1. Androgen Receptors

#### 3.1.1. Overall Survival (OS)

In order to demonstrate the correlation between the presence of AR and the OS, first we evaluate statistically the entire group of patients. Considering that the *p* value is > 0.05 (Table 2), we can conclude that the AR does not play a role for all the patients. 

**Figure 7:**
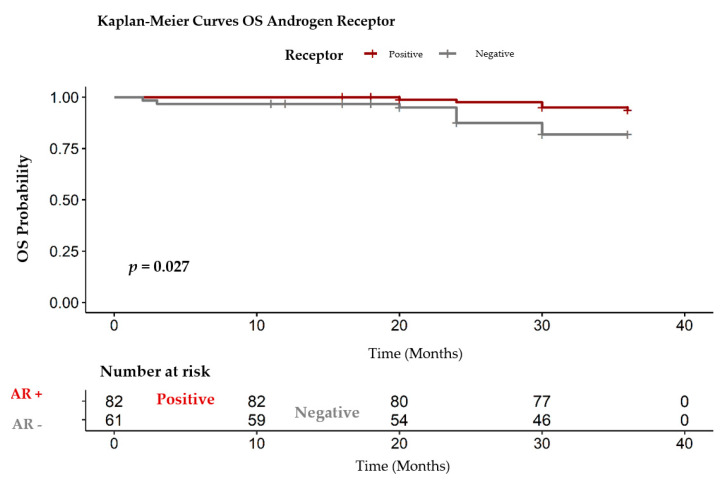
OS positive/ negative AR. OS: overall survival, AR +: androgen receptor positive, AR-: androgen receptor negative

To identify the pattern of relationship between presence or absence of the AR, we compared the patients with negative or positive AR (Figure 7). In oncology, the OS is estimated when the event, death in this case, occurs in the evolution of a patient. In our group of patients, this event appeared after 33.40 months in the group with AR negative and after 35.50 months in the positive group (Table 3). This correlation is statistically significant, with *p* = 0.027.

#### 3.1.2. Progression Free Survival (PFS)

**Figure 8:**
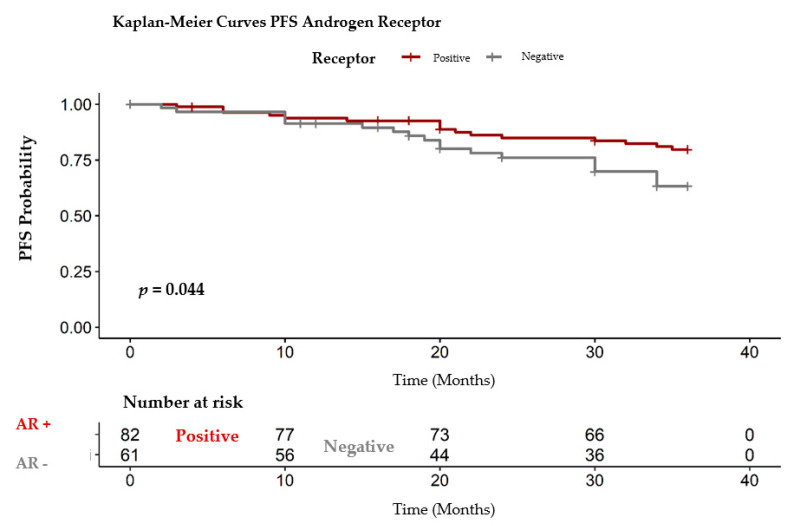
PFS positive/ negative AR. PFS: progression free survival, AR+: androgen receptor positive, AR-: androgen receptor negative

In oncology, the time to progression or the time until the onset of metastases is important. As the OS, the AR did not impact the PFS for the entire group of patients (*p* = 0.3830). When we compared the positive versus negative AR, we observed that the presence of AR has a favorable impact on the PFS (Figure 8), with *p*-value = 0.044, statistically significant. As shown in Table 2 the RMST PFS for the group with negative AR was 30.50 months from the diagnostic compared with 32.60 months for the AR positive group (Table 4). As shown in Table 4 the RMST PFS for the group with negative AR was 30.50 months from the diagnostic compared with 32.60 months for the AR positive group.

### 3.2. IHC Analysis

#### 3.2.1. Overall Survival (OS)

For the IHC analysis we divided the group of patients in four subcategories:

- Group I: triple negative breast cancer (TNBC) (*n* = 39)
- Group II: hormonal receptors negative, Her 2/neu positive (*n* = 22)
- Group III: hormonal receptors positive, Her 2/neu negative (*n* = 68)
- Group IV: hormonal receptors positive, Her 2/neu positive (*n* = 14).

**Figure 9:**
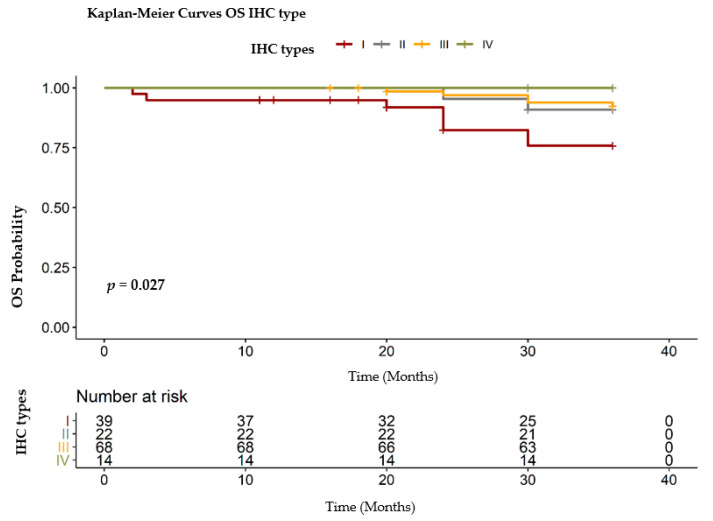
OS IHC distribution. IHC: immunohistochemistry, OS: overall survival, Type I: triple negative breast cancer, type II: hormonal receptors negative, Her 2/neu positive, type III: hormonal receptors positive, Her 2/neu negative, type IV: hormonal receptors positive, Her 2/neu positive.

In our group of patients, the lowest OS, so with the worst prognostic, was found in the TNBC group (Figure 9). Both the RMST (32.30 months) and the event percentage (20.51%) were significantly lower in this group of patients (log- rank test: χ^2^ = 9.20, degrees of freedom = 3, *p* = 0.0270). The better outcome, with no events, was achieved in the group with hormonal receptors (estrogen and progesterone) and her 2/ neu positive (Table 5).

#### 3.2.2. Progression Free Survival

Regarding the IHC and the PFS, in our study we did not find a correlation between the IHC subtype and the PFS. The value of the log- rank test: χ2 was 5.80, degrees of freedom = 3 and the *p*-value was 0.027, so statistically insignificant.

## 4. Discussion

This study confirmed that breast cancer is a complex disease comprised of distinct biological subtypes with diverse natural history which are increasingly recognized as presenting a varied spectrum of clinical, pathologic and molecular features with different prognostic and therapeutic implications.

Our institution is a large hospital in a metropolis city where patients are predominantly from urban areas. This may be a weakness of the study because we are often faced with patients with easy access to medical support. Moreover, the presence of all medical specialties gives the possibility to administer chemotherapy regimens safely, regardless of the associated pathology. Whenever necessary, evaluation of other specialties was requested (such as radiotherapy, plastic surgery, cardiology or anatomo-pathology) and all decisions were taken in the multidisciplinary tumor board. In our clinic, special attention is paid to patients with comorbidities. Each case is discussed with the specialized doctor together with detailed research of the specialized literature [40,41,42].

The routinely testing of other asymptomatic diseases is mandatory in our clinic. First and foremost, the assessment for both sexually transmitted diseases and screening for other types of cancers was performed for all patients [43].

Adherence to the treatment is very important to ensure the optimal therapeutic response. All the efforts were made to avoid delay, dose reduction or discontinuation of treatment. Even delay that has no relation to toxicity (legal holidays, other personal issues) was managed so that chemotherapy could be administered exactly when necessary. However, in about 10% of patients dose reduction or discontinuation of one of the cytostatic was required.

We assessed AR expression in 143 BC patients of different ages and with localized tumors and we found that this single marker was effective at predicting the evolution of the patients. This marker may impact both the OS and the PFS, if it is positive. Notably however, this association was not significant when we compared the entire group of patients. The OS was improved with 2.1 months in the AR positive arm, compared with the AR negative group.

A potential limitation of this study is the absence of the same treatment for this cohort, including the fact that treatment algorithms are likely to have been inconsistent over the six years’ time frame of this study. In addition, we have some patients with delayed scheduled treatment, so with less dose intensity and some patients with adverse events (anemia, febrile neutropenia, hypersensitivity reactions) for which it was necessary to postpone the treatment, to adjunct chemotherapy treatment or to interrupt it.

The chemotherapy regimens used in the study were delivered according to the international protocols in force, published by European Society for Medical Oncology (ESMO) or by National Comprehensive Cancer Network (NCCN) at the time of presentation in our clinic. About 10% of patients reported grade 3 or 4 adverse events and required delay or discontinuation of treatment. The principal adverse event was neutropenia, primary prophylaxis of neutropenia fever was always preferred in the presence of intermediate overall risk after taking into consideration all the patients risk factors. In the case of febrile neutropenia, we pay special attention to harvesting all peripheral smears and to isolating the patients in order to promptly establish an antibiotic therapy [44,45].

Few studies have tested the impact of the presence of androgen receptors (AR) on the BC cells and the results were gathered in meta- analysis. Kim et al. suggested that AR expression benefited both PFS and OS (OR, 0.44, 95% CI, 0.26–0.75; OR, 0.26, 95% CI, 0.12–0.55, respectively). Special attention was paid to the triple negative BC patients (TNBC), taking into account the severe prognosis and the lack of therapeutic approaches. Study by Qu et al. found that AR positive was beneficial to PFS in the TNBC subgroup (HR 0.40, 95% CI 0.23–0.69), but not for OS (HR 0.90, 95% CI 0.61–1.32) [46,47].

AR may therefore be an interesting tool to select patients for clinical trials, using this marker for prognostic or as a targeted therapy. Moreover, there are studies in which this marker was used as a surrogate for tissue biopsies using imaging positron emission tomography with different radiotracers. In a study publish in 2017 by Venema et.al metastatic BC patients with ER positive were enrolled irrespective of the AR status. The purpose of the study was to find out if it is a concordance between flour-dihydro-testosterone and 8F-fluoroestradiol PET and tumor AR and ER expression measured by immunohistochemistry. The authors conclude that it is a correlation between the radiotracers and the hormone expression and this technique may be used especially for biopsy associated sampling errors [46,48,49,50].

Although a clinical threshold that determines AR positively and negativity for BC patients has not been widely established, in this trial we consider that a positive result is more than 1%. We evaluated the impact of the expression and the absence of those receptors. Routine testing along with the classical triad IHC markers for BC appears to be an important prognostic factor for those patients.

The use of AR as a potential therapeutic target in breast cancer has yet to be established due to the difficulties in both the identification of the patients who might be benefit from AR-targeted therapies and the development of the right combination of therapeutic agents based on AR targeted therapies [9,14,24].

According to our data, AR was present in 57% of our patients. The presence of those receptors was statistically significantly correlated with an improvement of overall survival and progression free survival. These findings confirm that AR may be used as a prognostic factor and further studies are necessary for developing therapeutic targeted therapies. Till now, a few drugs were tested in BC patients with promising results; with the targeted population especially triple negative breast cancer. Furthermore, medical IoT (Internet of Things) enables continuous monitoring of patients, enabling remote gathering of results during their treatment, which is especially useful in case of pandemic crises when access to medical facilities is difficult [30,51].

The role of androgens in female BC seems to have dual effect, both pro- and anti- tumorigenic roles have been reported. Blood androgens are ligands which bind the membrane AR, who is internalized in cytoplasm and subsequently transported to the nucleus where it exerts the effects. The activation of targeted gene transcription leads to mRNA synthesis and subsequent protein synthesis, which finally determine cell proliferation, angiogenesis, cell division and differentiation. Without the ligand, the unbound AR is found in cytoplasm in a complex together with heat shock proteins. Given these data, we consider it is important to correlate the blood androgen levels with the positivity of the AR, moreover the association between increased androgen levels and high BC risk in pre- and postmenopausal patients. We consider that this aspect may be taken into account for the screening of BC [52,53,54].

In our clinic, we admit and deliver oncologic therapy only for patients with complete staging of the cancer disease. In this study, first and foremost, the tumor description with a complete histological and IHC report was mandatory. In order to demonstrate how hormone receptors may influence the prognosis in our group of patients, we divided the patients in four subgroups, according to the IHC results. We conclude that the TNBC molecular profile may have a negative impact on overall survival (32.30 vs. 36 months, *p* = 0.02) compared with both hormonal and Her2/neu positive BC patients. In the group of patients with positive ER, PR and Her 2/neu, the OS reached the maximum follow-up period. These expected survival differences have meaningful implications for communication and care decisions between providers and their cancer patients. This subclassification should, however, be complemented with the many other important prognostic variables for the individual such as age, tumor size, lymph node status, comorbidity, and adjuvant therapy.

While our study has the potential limitation of being retrospective and having a relatively small number of subjects, it has the advantage of consistency in immunostaining results. Using both biopsy and mastectomy specimens, that had been immediately fixed after sample collection, all processing steps and evaluations were performed in a single institution, an important feature of our study given that inconsistencies in the assessment of AR among institutions have yet to be resolved in order to ensure an accurate prognosis. Another important aspect is that the specimens were collected before receiving any oncological treatments.

## 5. Conclusions

The key strength of this study is the investigation of well characterized diagnosis BC samples this allowing an objective assessment of androgen receptor markers for prognostication of the BC. PFS and OS between groups of patients with different levels of each biomarker have been compared using log-rank tests with a 5% level of statistical significance. The group of patients with positive AR had a better OS and PFS. Correlated with the observation that TNBC had the poorest outcomes of BC patients, further studies are needed in order to establish which biomarkers in which cases can be used as prognostic factors.

New research studies along with advanced technological methods have shed light into the role of the AR in BC. Undoubtedly, the significance of the AR is getting clearer, as AR function and prognostic value had been elucidated over time. The proper identification of AR-partners and AR-target molecules will facilitate the understanding of BC pathogenesis. Further studies are needed to establish in which molecular types and menopausal status the AR may impact BC prognostic.

Despite the use of chemotherapy and immunotherapy nowadays, mortality in BC patients, especially TNBC, remains high. Therefore, it is necessary to find new therapies and AR may be an option. Moreover, the assessed of the AR may be used for molecular diagnosis for unknown primary tumors, especially TNBC.

## Figures and Tables

**Table 1:** The distribution of AR+ patients.

IHC	AR ≥ 1%	Range
TNBC (*n* = 39)	31 (80%)	15–90
R (−), Her 2/ neu (+), *n* = 22	14 (63%	10–100
R (+), Her 2/neu (−), *n* = 68	34 (50%)	10–30
R (+), Her 2/neu (+), *n* = 14	10 (71%)	15–90

AR: androgen receptor, TNBC: triple negative breast cancer R (-/+): estrogen and progesterone receptor negative or positive, *n =* number of patients.

**Table 2:** The AR distribution.

Predictor	Coefficient	*p*-Value	HR (CI 95%)
Androgen receptor	−0.002	0.7360	0.99 (0.98–1.01)

HR: hazard ratio, CI: confidence interval.

**Table 3:** OS AR survival data.

Strata	Events (%)	RMST OS	Median Survival OS (CI 95%)
AR+	6.09	35.50	N/A
AR−	16.39	33.40	N/A

AR+: androgen receptor positive, AR-: androgen receptor negative, OS: overall survival, N/A: not applicable, RMST: restricted mean survival time OS.

**Table 4:** PFS AR survival data.

Strata	Events (%)	RMST PFS	Median Survival PFS (CI 95%)
AR+	19.51	32.60	N/A
AR−	31.14	30.50	N/A

AR+: androgen receptor positive, AR-: androgen receptor negative, PFS: progression free survival. RMST: restricted mean survival time PFS, N/A: not applicable.

**Table 5:** OS IHC survival data.

Scheme 95	Events (%)	RMST OS	Median Survival OS (CI 95%)
TNBC	20.51	32.30	N/A
R (−) & HER 2 (+)	9.09	35.20	N/A
R (+) & HER 2 (−)	7.35	35.40	N/A
R (+) & HER 2 (+)	0.00	36.00	N/A

TNBC: triple negative breast cancer, R (−) & HER 2 (+): hormonal receptors negative, Her 2/neu positive, R (+) & HER 2 (−): hormonal receptors positive, Her 2/neu negative, R (+) & HER 2 (+): hormonal receptors positive, Her 2/neu positive, OS: overall survival, RMST: restrictive mean survival time OS, N/A: not applicable.

## Data Availability

The data published in this study was not report in any articles.
